# Decision-making about clinical trial options among older patients with metastatic cancer who have exhausted standard therapies

**DOI:** 10.1016/j.jgo.2022.01.012

**Published:** 2022-02-04

**Authors:** Mazie Tsang, Rebecca J. DeBoer, Sarah B. Garrett, Daniel Dohan

**Affiliations:** a Division of Hematology/Oncology, University of California San Francisco, 505 Parnassus Avenue, Room M1286, Mailbox 1270, San Francisco, CA 94143, United States; b Institute for Health Policy Studies, University of California San Francisco, 490 Illinois Street, San Francisco, CA 94158, United States

**Keywords:** Decision-making, Geriatric oncology, Clinical trial enrollment, Phase 1 trials, Older adults, Advanced cancer, Patient deliberation

## Abstract

**Objectives:**

Older adults under-enroll in early phase cancer clinical trials. There are limited data on their trial experiences, which hampers our ability to understand potential reasons and responses to under-enrollment. We aimed to explore older adults’ experiences and deliberations with phase 1 trials.

**Materials and Methods:**

We analyzed 101 in-depth interviews with 39 adults (average 2.6 interviews per participant) about their experiences with phase 1 trials. All respondents were ≥ 65 years and had advanced cancer. Interviews lasted 60–90 min and were audio-recorded, transcribed, and analyzed to identify respondents’ understanding of clinical research, perceptions of early phase trials, and experiences with enrollment.

**Results:**

Clinical trial participation was an interactive process that unfolded over time. Older adults relied on ongoing guidance and discussion with their oncologist to navigate the process. Respondents were generally interested in life-prolonging therapies, including enrollment in early phase clinical trials, but did not necessarily state this explicitly to their oncologist. While respondents did not mention age as a limitation to trials participation, participants age > 70 were less enthusiastic about participation and more often discussed their quality of life and weighed benefits of trial participation in the context of their remaining months of life.

**Conclusion:**

Early phase clinical trial enrollment is complex, and older adults rely on their oncologist to navigate this process. Acknowledging this complexity through shared decision-making may ensure that older adults have appropriate opportunities to enroll in early phase clinical trials and guard against inappropriate under-enrollment.

## 1. Introduction

Older patients have historically been excluded from or under-enrolled in cancer treatment trials relative to their numbers [1]. Systematic under-enrollment of older adults may diminish the utility of trial results or lead to potentially harmful extrapolation of results derived from younger populations [2]. It also raises ethical concerns regarding equity [1]. Barriers to clinical trial enrollment of older adults include ageism bias among oncologists, stringent eligibility criteria, concerns about toxicities, patient beliefs and understanding, and caregiver burden [3]. Trial design—including age limits, exclusion of patients with multiple comorbidities, non-specific functional status criteria, and generic organ function criteria—may systematically limit access to trials by older adults [4]. Additionally, older patients may prefer to avoid trial participation or may be unable to make informed, voluntary decisions to participate due to frailty associated with advanced age [5]. Data on how these factors shape the experiences of older adults as they navigate trial enrollment are limited, hampering our ability to develop strategies to improve older adults’ equitable and appropriate enrollment in cancer clinical research.

The phase 1 trial enrollment process provides a revealing context in which to examine issues of older adults’ equitable and appropriate enrollment in trials. Historically, phase 1 trials have been used to evaluate toxicities and identify the maximum tolerated dose of a treatment, and clinicians and trialists have considered older adult representation to be scientifically unnecessary and high risk [5]. At present, however, phase 1 trials are increasingly reporting response rates and measurements of clinical benefit, suggesting possible therapeutic intent [6]. Given evolving perceptions of the potential for benefits and risks of early phase trials, the American Society of Clinical Oncology (ASCO) has encouraged improvements in the patient education and informed consent process to ensure participants’ understanding about phase 1 trials [7].

In the standard model of phase 1 clinical trial enrollment, the oncologist invites a patient to consider a trial when standard therapy is no longer available. Qualitative data from the Cancer Patient Deliberation Study, however, show that contemporary early phase trial initiation occurs prior to this point [8,9]. Patients and oncologists often have conversations to set the stage for trial enrollment long before standard therapies are exhausted, and they evaluate eligibility in an attempt to secure a trial seat before consent is formally given [8]. These conversations occur in two stages: 1) setting the stage for trial participation through discussion between patients and their oncologists about the possibility of trial enrollment and 2) securing a seat in a clinical trial, which involves navigating the complex logistics of finding an open trial and initiating enrollment [8]. There remains a gap, however, in understanding the unique experiences of older adults as they navigate contemporary phase 1 trial opportunities. Qualitative research can help clarify how to improve equitable and appropriate access to early phase clinical trials by older adults.

In this study, we examine the early phase trial enrollment experiences of older adults who were part of the larger Cancer Patient Deliberation Study. Our objective was to understand how older adults think about and access early phase trials, navigate power dynamics, and evaluate other factors that might be involved in the enrollment process. We present these data using the two-stage model framework previously described in the Cancer Patient Deliberation Study [8].

## 2. Patients and Methods

### 2.1. Study Design and Recruitment

Data for this study are drawn from the Cancer Patient Deliberation Study, which used comparative ethnography of 96 participants to examine the early phase trial experiences of patients with advanced cancer at two academic medical centers [8]. Participants in the Cancer Patient Deliberation Study were diagnosed with stage IV cancer, as confirmed by an independent review of medical records and their treating oncologist. Treating oncologists identified participants who nearly exhausted standard therapies and were potential candidates for enrollment in an early phase clinical trial. Additional inclusion criteria included being cognitively intact and able to effectively communicate in English. This secondary analysis included a subset of all patients in the original cohort who were age ≥ 65 at the time of the study. The University of California San Francisco Institutional Review Board has approved this study.

### 2.2. Data Collection and Management

Trained fieldworkers collected data at 8 multi-disciplinary clinics at two academic centers (in the Pacific and Midwest regions) via observation of up to four clinic visits, a survey administered to patients and caregivers, and multiple 45-to 90-min in-depth, semi-structured interviews using open-ended questions with patients and their caregivers. Interviews addressed their clinic visits, medical history, decision-making process, knowledge of clinical trials, social support, socioeconomic background, and spirituality. Patient interviews were digitally recorded, professionally transcribed, and entered into ATLAS.ti qualitative data analysis software (version 7.5, Berlin, Germany). A codebook was developed based on literature and ongoing insights gained during fieldwork. Data were coded by three trained coders, and 20% of the data were double-coded in order to assess consistency and enhance rigor. Inter-coder reliability kappa scores were above 0.80, indicating high agreement [10]. After coding all data, the research team used the coded database to develop an analytical case summary for each participant in the study. These case summaries documented key clinical and decision-making events in the context of the patient’s social environment and in relationship to key themes of the parent study [8]. Data also included comments from any caregivers who were present with the participants at the time of an interview. Additional information about study design, methods, and procedures were previously published [8,10–12].

### 2.3. Data Analysis

In the present study, we re-analyzed semi-structured interviews with 39 patients (and caregivers when present during the interview) who were ≥ 65 at the time of enrollment. We did not include clinic observation or survey data. DD and MT (trained by DD) conducted thematic analysis [13]. The analysis involved DD and MT reading and coding 2–3 participants’ transcripts at a time to identify, discuss, refine themes, and resolve any differences in interpretation. DD and MT developed a secondary coding system by adding codes for topics that emerged in a review of relevant literature. We repeated this process across multiple rounds of transcripts. We determined that thematic saturation was achieved when themes were repeated in the data and further analysis yielded no revisions or additions to the themes [14]. We achieved thematic saturation after twelve transcripts for patients >70 and fifteen transcripts for patients aged 65–70. We coded the full corpus of transcripts to capture the themes across the full breadth of the sample. Throughout this process, DD and MT discussed codes and analyses with a third author (RD) to evaluate and affirm consensus. We used the case summaries from the parent study to affirm consensus and provide information about important clinical and decision-making events. We contextualized this analysis by drawing on the two-stage framework of early phase trial participation (Table 1) developed by Garrett et al. [8] Our findings are themes we identified in the accounts of older adults as they navigated the two stages of early phase clinical study participation. Patients were defined as having initiated a trial if they were referred to a dedicated phase 1 clinic at either the Pacific or Midwest site or if they reviewed the clinical trial consent form at the Midwest site but had not yet started on the trial [11]. We considered a participant to have enrolled in a trial if they signed the consent form [8,11]. We also identified patients’ disposition at the end of the study.

## 3. Results

Patient characteristics are summarized in Table 2. Most patients were college graduates: 62% held at least a college degree and 41% held an advanced degree. The participants experienced a heterogeneous set of outcomes related to trial participation (Table 3). We identified three themes, which we discuss below using the two-stage framework. We showed that oncologists played a central role in leading patients through the early phase trial enrollment process. We also inductively found subtle differences between the experiences of patients who were age > 70 (“>70 subgroup”) compared to age 65–70 (“65–70 subgroup”).

### 3.1 Patient Experiences while Setting the Stage

During this stage, patient and clinician deliberations focused on whether the patient was interested in early phase trial participation and whether the oncologist supported the patient’s participation. There were two steps in this stage that occurred in no particular order. One of the two steps was to identify older adults’ interest in cancer clinical trials:

“…Both my wife and I wanted to get into this clinical trial very badly and get it going as soon as possible. And we have never wavered from that approach since we started talking about clinical trials months ago.” –Patient 14.

The other step was that oncologists recommended or expressed support for clinical trial enrollment. This happened prior to older patients’ exhausting standard therapies:

“There was a clinical trial that was showing great results and he really felt confident that mom would be a really good candidate [for] the clinical trial and we were definitely all for it.” —Patient/caregiver 27.

First theme: patients deferred to their oncologist to gauge their interest in clinical trials and discuss trial availability. Although some patients researched clinical trials, patients depended on their oncologists to make decisions because they expressed that their oncologists were more knowledgeable about cancer treatments and best options:

“He [oncologist] makes the same decisions I do. [Laughter] And if he has to make a different kind of decision, when he explains it to me, I know he’s right, you know? I’m not thinking, ‘What?’” —Patient 29.

The oncologists’ general support for enrollment played a central role in whether a patient explored trial participation. Patients deferred to their oncologist for education on the next best steps in treatment, help with decision-making, and trial possibilities:

“My decision-making is basically what [my oncologist] suggests. I do what the professionals say.” –Patient 33.

A main reason for this deference to their oncologist was that the research and decision-making could be overwhelming:

“I tried to look [at research] and they were scary… I read some but I stopped reading.” –Patient 35.

Second theme: there were modest differences between how participants age 65–70 and ≥70 approached trial exploration with their oncologists. Participants in the 65–70 subgroup tended to come to the academic center specifically to discuss trials after heavily researching potential clinical trial options or speaking with their community oncologist. Patient 9, for example, set up an online search alert for articles involving potential new therapies for his cancer. Some in the 65–70 subgroup saw early phase trials as an expected part of their therapy, whereas others saw it as a last resort:

“I researched places because we know that his only hope is doing trials. So I researched the trials to find out who was doing them, who was somewhat successful; where could we go” –Patient/caregiver 8.

The >70 subgroup were more deferential to their oncologists regarding next steps in treatment. They were less likely to explicitly seek out and discuss trial enrollment or distinguish phase 1 trials from standard of care:

“I just thought it was part of the treatment; it was what a doctor should do.” –Patient 33.

The >70 subgroup, in comparison to the 65–70 subgroup, did not express the same degree of hope in clinical trials:

“My understanding is that…this cancer was not curable but that these clinical trials could possibly reduce this illness to a chronic illness, manageable state. …. Should a trial turn out to be a miraculous cure, which nobody really expects, but should that be the case, then bonus.” –Patient 30.

The >70 subgroup were also more vocal about weighing the time commitment required for a clinical trial against the potential clinical benefit, which was not explicitly mentioned by the 65–70 subgroup:

“On the other hand, it’s a time commitment that is extraordinary…Do I really want to spend my time in your waiting room?” –Patient 28.

Participants with altruistic goals of promoting scientific advancement through trial enrollment were among the >70 subgroup:

“…I’ve participated in several clinical trials and I like doing things that help people learn. If I could help them find a cure for cancer, that would be absolutely incredible…. Hopefully they learn from my participation.” –Patient 15.

Lastly, the >70 subgroup were more explicit about their desire for a clinical trial to maintain or enhance their quality of life:

“[My goal is] To extend it with quality, not extend the life and be miserable. No, no, no. I keep telling my sons and my grandsons, ‘If I’m going to be on the respirator for a year, no. Knock it off.’ If I not be able to be on my feet, I prefer to be on my knees for a short period of time.” –Patient 23.

### 3.2. Patient Experiences when Securing a Seat

During this second stage, which occurred after or alongside the first stage, clinicians and patients identified an open seat on a clinical trial, affirmed patient eligibility, and established informed consent:

“We were looking at clinical trials…I was first or second on a waiting list, but it closed out with the people that were on it… And that’s when she decided to write the appeal… It’s pretty forbidding when you start looking at the stuff that they send you, ‘cause I had all the application forms for it and it’s pages and pages…” –Patient 28.

To be evaluated for eligibility, older adults needed to have the wherewithal to navigate a battery of tests:

“They said before you can get in the study…you have to have bone density – You have to get all these tests.”—Patient 34.

Third theme: patients heavily relied on their oncologists for guidance in setting the stage for enrollment and securing a seat to participate. After joining a trial, participants continued to rely on their oncologist to navigate the complex early phase enrollment process and make ongoing assessments of whether trial participation was appropriate. For example, one patient trusted her oncologist to withdraw her from a clinical trial before starting treatment:

“[My oncologist] presented us with a clinical trial… Just before we were ready to start it, he decided he didn’t want me in there. He didn’t want to take that chance. So he pulled me out of the trial and then I went into a whole series of chemo.” –Patient 32.

Despite their interest, only a subset of eligible patients in both subgroups eventually initiated a clinical trial (Table 3). Our findings were limited to the patient interviews. Some patients were then ineligible to enroll, e.g., they had prior therapy or did not meet biologic parameters (such as having elevated liver enzymes). The participants did not mention that their age, functional status, and comorbidities were reasons for being ineligible for a trial. Overall, they trusted their oncologist to determine their eligibility and appropriateness for trial enrollment.

Like the first stage, there were subtle differences in how the >70 subgroup navigated trial initiation. They spoke in less depth and detail about their oncologist’s judgement about clinical trial participation and whether a trial represented a reasonable alternative to the standard of care options. They made briefer comments about whether their oncologist told them they were eligible for a trial. This contrasts with the 65–70 subgroup, who were more detailed and specific about their eligibility and referenced their involvement in the evaluation process:

“They were still recruiting patients so we came roaring down here... ‘Cause they had three or four days of pretesting to see if you were even eligible.” –Patient 2.

## 4. Discussion

Oncologists often invite a patient to join an early phase study only when the patient exhausts standard therapies [15,16]. Deliberations about trial participation, however, begins before this invitation is extended. Qualitative research shows that early phase trial enrollment is a process that unfolds over time. Improving study recruitment and enrollment thus benefits from analyses that begin before the moment at which a patient reviews and signs consent forms.

This ethnographic study described the experiences of older adults with advanced cancer as their available therapeutic options diminished. We used a two-stage model of early phase trial enrollment to distinguish activities that set the stage for trial participation from those involved in securing a seat on a trial. This model has proved useful in capturing the dynamic process of early phase trial enrollment [8].

Prior studies have drawn lessons about how to improve early phase trial enrollment processes from experiences of younger patients [17,18]. A central question in this study is whether the experiences of older adults with advanced cancers could provide further insights. Our analyses showed that older patients generally relied on their oncologist for guidance and that they navigated the process somewhat differently depending on their age. We found that the experiences of older adults held lessons for oncologists about how to educate and obtain informed consent during trial recruitment and enrollment. In particular, the >70 subgroup viewed early phase clinical trials in terms of their remaining time, quality of life, and personal values. Oncologists could foster appropriate recruitment of older adults onto phase 1 trials by focusing on setting the stage, i.e., when and how they discuss clinical trials with their older patients [19].

Oncologists want to ensure that patients of all ages are appropriately included in phase 1 trials. To achieve this requires an examination of the stage-setting process, e.g., whether older adults had an opportunity to learn about and discuss trial participation, as well as an examination of securing a seat, e.g., whether older adult enrollment was impeded by clinical eligibility criteria. Some studies showed that setting the stage was the primary problem [20]. In a study of 577 older patients who were considered for 37 trials, one third of eligible older adults were not invited to participate in clinical trials [21]. Other studies showed that oncologists set the stage by deciding which patients were good candidates for clinical trial enrollment based on trial availability, eligibility, and patient performance status [22], or by their ability to adhere to research protocols [23]. In another study, older patients were also less likely to actively seek out trials, although they were willing to participate in a clinical trial if their oncologist recommended it, and it might help them feel better [24]. These findings are in contrast to younger patients who were more likely to inquire about trial availability and actively enroll in cancer clinical trials [25]. Other studies have found that securing a seat on a trial was difficult—clinical trials were either unavailable, or older adults were ineligible for available trials [20,21].

Like prior studies, we found that interactions in setting the stage were paramount in phase 1 clinical trial recruitment. Older patients with advanced cancer are accustomed to conversations about treatment options, and they rely on their oncologist to guide them. Often, oncologists or patients discuss trial participation before standard treatment options are exhausted. Oncologists might express general support of clinical trial enrollment or support patients who raised the prospect of a study. In any case, older patients deferred to their oncologist. Therefore, oncologists who wish to support appropriate trial recruitment of older adults should be sensitive to the importance of conversations about trials. Having these conversations early in disease progression may be important in setting the stage for future trial participation. Such conversations may be initiated by patients, and oncologists may also consider whether to discuss early phase trials as an option even at an early point in the treatment trajectory of an older adult with metastatic cancer.

Our ethnographic data show early phase clinical trial enrollment is an interactive, deliberative process. We highlight the potential value of an individualized and patient-centered approach to these deliberations. Older adults may see phase 1 trials as an extension of treatment. Traditional motivations to join such a trial, e.g., altruism or advancing science, may take second place to treatment considerations when joining an early phase trial. These data provide insights into different strategies that oncologists can use as they frame phase 1 trial participation for their patients as a consideration worthy of further deliberation.

Lastly, we showed subtle differences in the way that the >70 subgroup approach decision-making about early phase research. Participants age > 70 considered many factors when deliberating about early phase clinical trial enrollment—as older adults with advanced cancers age, they experienced a shift in how research would impact their quality of life and how they would like to spend their available time. They also were more likely to discuss early phase trials as experimental therapy, rather than placing a lot of hope in the trials. Despite the differences in how the >70 subgroup deliberated about trial participation, all older adults in our study looked to their oncologist to recommend the most appropriate next steps in treatment, including whether to enroll in a trial or pursue other options. As a result, oncologists should tailor their conversations with older adults to include quality of life, time commitment, and the role of phase 1 trials in the larger context of their disease course.

This study elucidated experiences of older adults who might be candidates for phase 1 trial enrollment. It avoids focusing on the moment of trial invitation when standard therapies are exhausted and a patient is considering whether to sign a consent form. A two-stage framework provides a more grounded description of how older adults experience the transition that occurs as standard therapies become clinically futile [8]. We found that older adults are generally open to participating in clinical trials if recommended by their oncologist, though they tend to more heavily weigh the trade-offs of potential wasted time and quality of life with increasing age. Other age-related factors cited in previous studies, such as frailty, safety concerns, and ageism, did not emerge as significant barriers in our study. Future work should evaluate how oncologists invite patients to enroll on early phase trials and how to design interventions that diversify enrollment of underrepresented groups.

### 4.1. Limitations

This study produced rich ethnographic data for examining the experiences of 39 older adults on early phase clinical studies. Our findings are based on data derived from patient interviews and do not include objective information about the participants’ cancers or treatments. In transcripts in which caregivers were also present at the interviews, we did not analyze patient and caregiver comments separately or evaluate for alignment within patient-caregiver dyads. It is possible that some dyads were more aligned than others, and this could have influenced decision-making. Lastly, having been based in two academic medical centers, these findings may not generalize to other phase 1 trials settings and were not representative of a larger population. Participants were largely white, English-speaking, college educated, and of high socioeconomic status. While these characteristics may be common among patients at the academic medical centers where early phase trials often take place, our results cannot speak to how the process unfolds among diverse patient populations.

## 5. Conclusion

Our data elucidate the motivations that lead older adults to join early phase cancer trials and the critical role of oncologists in their participation. Ethnographic data suggest that therapeutic benefit – alongside altruism – motivates patients. These data also illustrate the important role oncologists play in setting the stage for older adults’ participation as patients and oncologists may consider or discuss studies long before standard treatments are exhausted. When setting the stage, oncologists should be aware of how older adults approach phase 1 studies and any ethical concerns that may arise. They should explain the risks, goals, and benefits to determine whether phase 1 participation is well aligned with their older patients’ goals and values, acknowledging that some patients might feel there are potential therapeutic benefits. This approach to early phase trial participation may enhance opportunities for enrollment among older adults and other groups currently underrepresented in research.

## Figures and Tables

**Table 1:** Two-stage framework of Phase 1 trial enrollment (Garrett et al., 2020).

Activity	Description
Stage 1: Setting the Stage	Involves deliberations about whether oncologists support early phase participation and whether the patient is interested.
Part 1: Oncologist Support	Oncologists determine whether a clinical trial is appropriate for the patient and whether there is one available.
Part 2: Patient Interest	Patients are open to or express interest in clinical trials.
Stage 2: Securing a Seat	Occurs after stage 1 or concurrently. Involves identifying an open trial, affirming eligibility, and obtaining informed consent.

**Table 2:** Patient characteristics of older adults age ≥ 65 in the cancer patient deliberation study.

Characteristic	No.	%
Age 65–70	22	56
Age > 70	17	44
Female gender	15	38
Ethnicity		
African American	3	8
Asian/Asian American	3	8
Hispanic	2	5
White	26	66
Other	1	3
Not answered	4	10
Disease site		
Breast	5	13
Gastrointestinal	11	28
Genitourinary	15	38
Lung	2	5
Melanoma	6	15
Education		
Less than a bachelor’s degree	13	33
College education	8	21
Advanced degree	16	41
Not answered	2	5
Annual household income		
<20 K	1	2
20 to <40 K	3	8
40 to 60 K	4	10
60 to <80 K	2	5
80 to 100 K	3	8
>100 K	17	44
Not answered	9	23
Relationship status		
Divorced/Separated	8	21
Married/Partnered	26	66
Widowed	2	5
Not answered	3	8

**Table 3:** Trial status of individual patients.

Patient	Age	Sex	Cancer type	Trial status^a^
1	65	F	Colon	Initiate
2	65	F	Melanoma	Not in a trial
3	65	F	Breast	Initiate
4	65	M	Prostate	Not in a trial
5	65	M	Prostate	Enrolled
6	66	F	Breast	Initiate
7	66	F	Pancreatic neuroendocrine	Not in a trial
8	66	M	Melanoma	Not in a trial
9	66	M	Prostate	Not in a trial
10	67	F	Breast	Enrolled
11	67	F	Kidney	Not in a trial
12	68	M	Prostate	Enrolled
13	68	M	Pancreatic	Not in a trial
14	68	M	Lung	Enrolled
15	68	F	Pancreatic	Not in a trial
16	68	M	Prostate	Not in a trial
17	69	M	Bladder	Not in a trial
18	69	M	Prostate	Not in a trial
19	70	M	Liver	Not in a trial
20	70	M	Melanoma	Not in a trial
21	70	F	Breast	Not in a trial
22	70	M	Prostate	Not in a trial
23	71	M	Colorectal	Not in a trial
24	71	F	Rectal	Not in a trial
25	71	M	Pancreatic	Not in a trial
26	71	M	Melanoma	Not in a trial
27	71	F	Melanoma	Not in a trial
28	71	M	Prostate	Not in a trial
29	72	F	Melanoma	Not in a trial
30	73	M	Esophageal	Initiate
31	74	M	Prostate	Not in a trial
32	75	F	Lung	Enrolled
33	75	M	Colon	Not in a trial
34	75	F	Breast	Not in a trial
35	78	F	Esophageal	Not in a trial
36	78	M	Leydig cell	Enrolled
37	78	M	Prostate	Enrolled
38	83	M	Prostate	Not in a trial
39	95	M	Prostate	Not in a trial

Initiate: Either (a) referred to a dedicated phase 1 clinic at either the Pacific or Midwest site or (b) reviewed the clinical trial consent form at the Midwest site but had not yet signed the consent form.

Enrolled: Signed the consent form.

Not in a trial: Did not initiate or enroll in a trial.

a Trial Status.
